# Referential vs. Non-referential World-Language Relations: How Do They Modulate Language Comprehension in 4 to 5-Year-Olds, Younger, and Older Adults?

**DOI:** 10.3389/fpsyg.2020.542091

**Published:** 2021-01-13

**Authors:** Katja Maquate, Pia Knoeferle

**Affiliations:** ^1^Psycholinguistics, Department of German Studies and Linguistics, Humboldt-Universität zu Berlin, Berlin, Germany; ^2^Einstein Center for Neurosciences, Berlin, Germany; ^3^Berlin School of Mind and Brain, Berlin, Germany

**Keywords:** real-time language processing, children, older adults, emotional faces, referential effects, non-referential effects, age differences, visual-world eye-tracking

## Abstract

Age has been shown to influence language comprehension, with delays, for instance, in older adults' expectations about upcoming information. We examined to what extent expectations about upcoming event information (who-does-what-to-whom) change across the lifespan (in 4- to 5-year-old children, younger, and older adults) and as a function of different world-language relations. In a visual-world paradigm, participants in all three age groups inspected a speaker whose facial expression was either smiling or sad. Next they inspected two clipart agents (e.g., a smiling cat and a grumpy rat) depicted as acting upon a patient (e.g., a ladybug tickled by the cat and arrested by the rat). Control scenes featured the same three characters without the action depictions. While inspecting the depictions, comprehenders listened to a German sentence [e.g., *Den Marienkäfer kitzelt vergnügt der Kater*; literally: “The ladybug (object/patient) tickles happily the cat (subject/agent)”]. Referential verb-action relations (i.e., when the actions were present) could, in principle, cue the cat-agent and so could non-referential relations via links from the speaker's smile to “happily” and the cat's smile. We examined variation in participants' visual anticipation of the agent (the cat) before it was mentioned depending on (a) participant age and (b) whether the referentially mediated action depiction or the non-referentially associated speaker smile cued the agent. The action depictions rapidly boosted participants' visual anticipation of the agent, facilitating thematic role assignment in all age groups. By contrast, effects of the non-referentially cued speaker smile emerged in the younger adults only. We outline implications of these findings for processing accounts of the temporally coordinated interplay between listeners' age-dependent language comprehension, their interrogation of the visual context, and visual context influences.

## 1. Introduction

Psycholinguistic studies have shown that action and social contexts can rapidly modulate participants' expectations and real-time language processing (e.g., action depictions between agents and patients, such as a princess punching a fencer, Knoeferle et al., 2005; a speaker's facial expression, revealing emotion, Carminati and Knoeferle, 2013; and voice characteristics, revealing age or social status, Van Berkum et al., 2008). The same is true for comprehender characteristics such as level of education (Huettig et al., 2011). Old age, as another comprehender characteristic, can impact language comprehension (e.g., Just and Carpenter, 1992; Kemtes and Kemper, 1999; DeLong et al., 2012), perhaps via cognitive decline (Just and Carpenter, 1992; Salthouse, 1996). Young age in turn modulates language comprehension, perhaps because of differences in competence or cognitive resources (e.g., Dittmar et al., 2008; Schipke et al., 2011). Many of these distinct context and comprehender effects on language comprehension and visual attention have been accommodated in an account of language processing [the social Coordinated Interplay Account (sCIA); Münster and Knoeferle, 2018].

We know too little, however, about context effects, also in relation to age variation to predict specifics of how rapidly (visual) context modulates comprehension across the lifespan. Consider an example: Action verbs (e.g., *to tickle*) can reference an event in the world or in a depiction (e.g., someone being tickled by someone else). To establish this referential world-language link, comprehenders must interpret the verb and apprehend the action. The referenced action can unlock associated information such as its agent. If so, then encountering a verb could—via reference to an action—cue whoever is doing the tickling before that person is mentioned. The link between this type of visual contextual information, i.e., seeing someone tickling someone, and situated language, i.e., hearing *She's tickling the kid!* is directly referential, in the sense that we can map the linguistic input directly onto the visual context.

By contrast, cueing an agent via a speaker's smile may be possible but is not grounded in accounts of reference (Jackendoff, 2002). Imagine a speaker smiles and says: *The kid tickled happily by…*. In principle, the smile signals happiness and a tendency to say something positive, prompting a listener to expect a positive sentence and event. That expectation is confirmed when the listener hears (*happily*) and notices a happy-looking grandma doing the tickling. Jointly, the speaker's smile, happy adverb, and the grandma's smile could permit a listener to anticipate the grandma as the agent. This relation between a speaker's smile, a happy adverb, and (in this case) the grandma's smile, which is not grounded in accounts of reference, presents an example of what we define as a non-referential world–language relation.

Do such distinct world-language relations, involving depicted actions vs. emotional facial expressions, affect comprehension similarly? The effects of actions in a scene, of gender stereotypes, of emotional facial expressions, of speaker accent, and a speaker's eye gaze all seem to occur similarly rapidly during comprehension (Chambers et al., 2002, 2004; Knoeferle et al., 2005; Dick et al., 2009; Carminati and Knoeferle, 2013; Holler et al., 2014; Rodríguez et al., 2016; Kröger et al., 2018; Jachmann et al., 2019; Xu et al., 2019).

Alternatively, do they modulate comprehension in distinct ways (Knoeferle et al., 2014; Kreysa et al., 2018; Knoeferle, 2019)—much like different sorts of linguistic knowledge are processed distinctly (Hagoort, 2003; Huettig and McQueen, 2007; Bastiaansen and Hagoort, 2015; Lapinskaya et al., 2016)? Initial evidence suggests that referential and non-referential world-language relations modulate comprehension and visual attention in distinct ways with referential relations eliciting more visual attention to objects than non-referential relations (Cooper, 1974).

A range of studies on distinct world-language relations (Knoeferle et al., 2014; Kreysa et al., 2018; Kröger et al., 2018) pitted action verbs and their referents against other cues and world–language relations (speaker facial emotion, role relations, speaker gaze, or a wiggle motion of a character). Unlike the case of an action verb denoting a depicted action, these other world–language relations were non-referential. Nonetheless, their effects on real-time sentence processing seemed rapid, incremental, and dependent on comprehender characteristics such as age and cognitive abilities.

To illustrate, online processing of written sentences can be distinctly modulated by verb-action compared with depicted role relations, and these effects varied with participants' verbal memory and visual-spatial abilities (Knoeferle et al., 2011; Experiment 1 in Knoeferle et al., 2014). Measuring ERPs in a picture-sentence verification paradigm, participants first inspected a clipart scene depicting two characters and an action between them (e.g., a gymnast punching/applauding a journalist; a journalist punching/applauding a gymnast). Next, participants read an English subject-verb-object sentence (e.g., *The gymnast punches the journalist*) in rapid serial visual presentation, which either matched the scene, mismatched in the action, in who was doing what to whom, or both (Knoeferle et al., 2014). Action-verb mismatches (vs. matches) elicited larger N400 mean amplitudes time-locked to the onset of the verb. Role mismatches (vs. matches) elicited an anterior negativity to *the gymnast* and a larger positivity to the early verb. The distinct mismatch effects suggested differences in the underlying mechanisms, a conclusion that was also supported by distinct correlations with participants' mean accuracy, verbal working memory, and visual-spatial scores.

Variation of such context effects by comprehender age (e.g., children vs. young adults vs. older adults) could reveal at what time in the lifespan what sorts of world-language relations are (vs. aren't) beneficial. DeLong et al. (2012) suggested that older adults derive expectations during comprehension in strictly linguistic contexts less well than younger adults (see also Federmeier et al., 2002, 2010). Uncovering age-dependent context effects could constrain predictions in accounts of situated language comprehension and inform accounts of contextual aids in language learning. Regarding lifetime variability of visual context effects, some findings suggest substantial variation in the implicated language comprehension processes (Trueswell et al., 1999), whereby the number of referents in context affected young adults' but not children's resolution of local structural ambiguity; others have argued for similarity of the processes and differences in the time course of visual context effects on language processing (Meroni et al., 2003).

Motivated by the reviewed results, we examined the effects of distinct world-language relations and of comprehender age in three visual-world eye-tracking experiments, and modeled the results in a detailed theoretical account of language processing. All three experiments examined to what extent verb-referenced depicted action events and an emotional facial expression of a speaker facilitate the anticipation of a depicted target agent. This target agent was the subject and agent in a non-canonical German Object-Verb-Adverb-Subject (OVAdvS) sentence. We chose depicted action events since verb-action relations are referential and can rapidly facilitate younger adults' and children's real-time thematic role assignment of unambiguous German OVS sentences (cf. Knoeferle et al., 2005; Zhang and Knoeferle, 2012; Münster, 2016; Kröger et al., 2018). We chose an emotional speaker face as a non-referential cue. It can guide attention to an entire event depiction during the comprehension of subject-first clauses (Carminati and Knoeferle, 2013), lending some credence to it also cueing the agent within an event during comprehension of object-first sentences.

**Figure 1:**
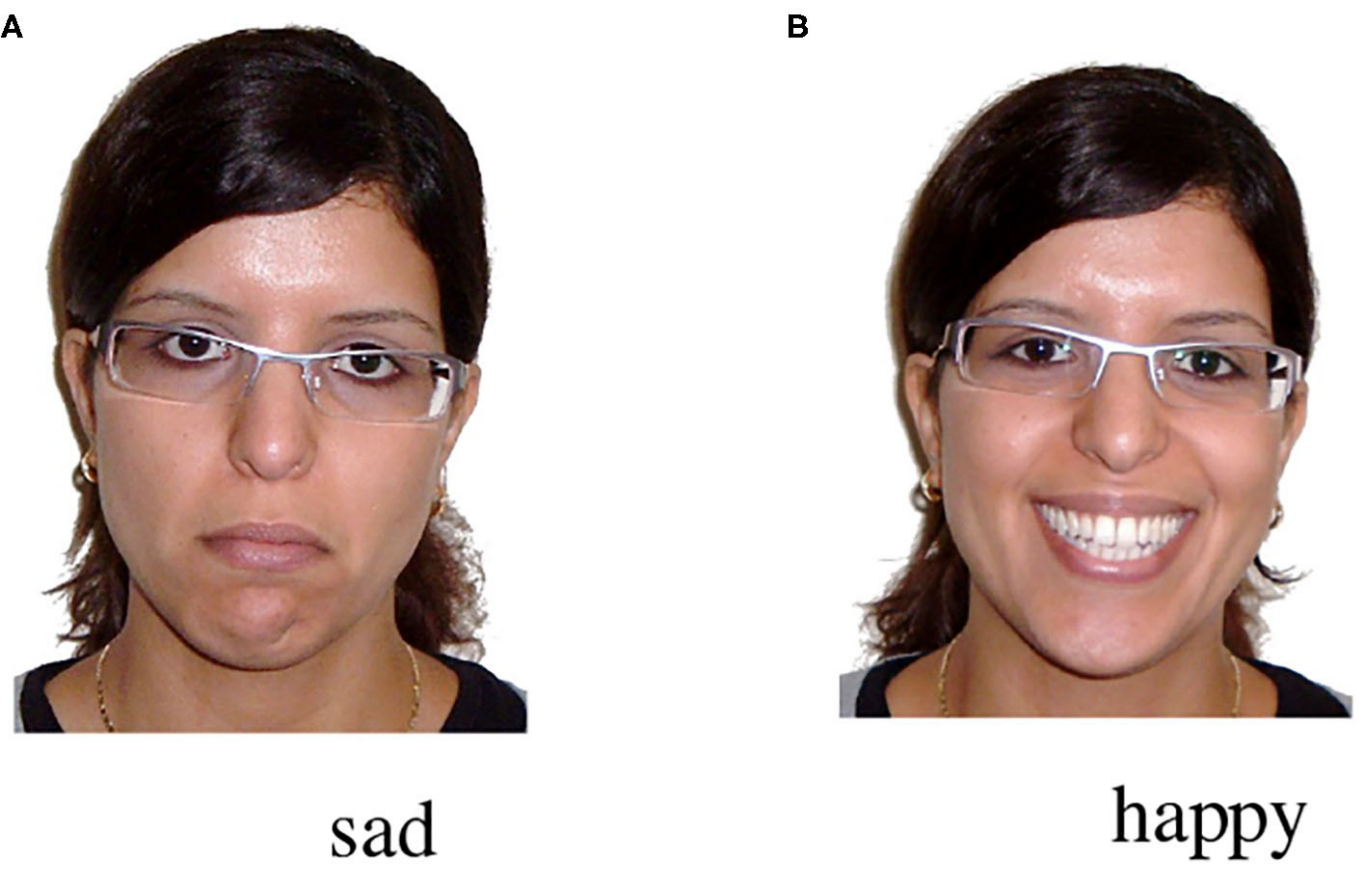
Snapshots from the prime face videos: **(A)** sad and **(B)** happy.

In each experiment, participants first saw a video of a speaker smiling broadly or looking sad (see Figure 1). Next, participants inspected a clipart scene depicting three characters: a happy-looking agent, a neutral-looking patient, and grumpy-looking competitor. Shortly after the onset of the clipart scene, they listened to a positively valenced OVAdvS sentence (i.e., *Den Marienkäfer kitzelt vergnügt der Kater*, lit. transl.: “The ladybug _NP1:Patient−Object−Acc_ tickles _verb_ happily _adverb_ the cat _NP2:Agent−Subject−Nom_”).

**Figure 2:**
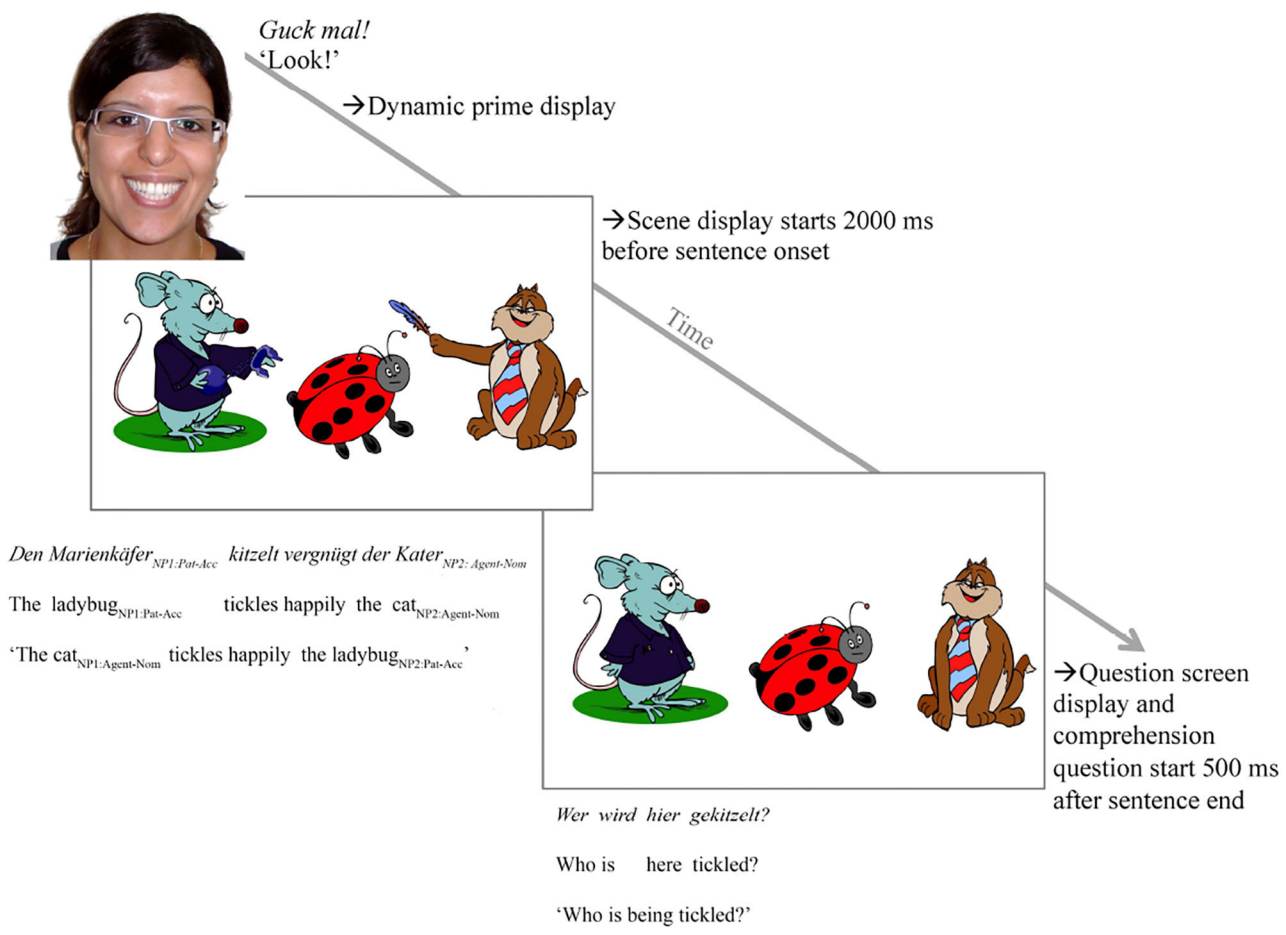
Experimental procedure showing a trial in condition a (action) (see Table 1). Note that the figure shows the happy speaker face only (“->Dynamic prime display”); speaker face was, however, manipulated and participants saw a sad speaker face for the sentence-mismatching negative prime face condition. The figure further shows the clipart scene for the depicted action condition only (“->Scene display”); for the no action condition, the characters were identical but no actions were depicted, just like in the “->Question screen” (see Table 1 for details on the design).

The smiling (but not the sad) speaker face matched sentence valence. In the clipart scenes, the competitor looked grumpy, mismatching the speaker's smile. However, the agent (here: a cat, see Figure 2) always smiled, linking to the speaker's positive but not her negative facial expression and the positively valenced sentence, in principle permitting anticipation of the agent. For instance, having seen a speaker smile and hearing *The ladybug is tickled happily …*, participants might look for a happy tickling action and thus anticipate the cat agent doing the tickling before it is mentioned. Such anticipation would indicate that the manipulated world-language relations can alleviate processing difficulty associated with OVS sentences (Matzke et al., 2002; Kamide et al., 2003; Scheepers and Crocker, 2004; Knoeferle et al., 2005).

The verb in the sentence matched the action of the agent (but not of the competitor), thus also permitting participants to anticipate the agent when actions were depicted. For half of the experimental trials, actions were depicted; for the other half, only the three characters were visible. We use the shorthand “depicted actions” in the article as a term for action depictions in a clipart scene.

This design permits us to directly compare the effects of these two world-language relations for differences and similarities. By examining effects of these world-language relations in younger adults (experiment 1), children (experiment 2), and older adults (experiment 3), we gain insight into variability of such context effects across the lifespan.

## 2. Experiments

### 2.1. Method

#### 2.1.1. Participants

Forty monolingual German speakers took part in each experiment (younger adults, exp. 1: 18–30 years, *M* = 23.75, *SD* = 3.6; children, exp. 2: 4–5 years of age, *M* = 4.37, *SD* = 0.5, and older adults, exp. 3: 60–90 years, *M* = 67.1, *SD* = 6.1). Participants responded to flyers and announcements in and around the university and city of Bielefeld. Children were tested in Kindergartens; older and younger adults were tested in the eye-tracking laboratory. All had normal or corrected-to-normal vision and hearing. Adult participants received a monetary reward and children a small toy and a participation certificate. We obtained ethics approval from the Bielefeld University Ethics Committee (Vote 2013-007).

#### 2.1.2. Materials and Pretest

##### 2.1.2.1. Prime face videos

The speaker's emotional facial expression (the prime face) was presented as a video in which a woman changed her facial expression from neutral into a broad and open smile/sad expression after 1,300 ms (video duration: 5,500 ms). The last frame of the video was thus either positive or negative (see Figures 1A,B).

The woman's face had been chosen on the basis of a previous 9-point scale rating study on 15 faces (see DeCot, 2011; Carminati and Knoeferle, 2013, unpublished Master thesis, *N*_participants_ =18, *M*_age_ = 24.7, *SD*_age_ = 2.74). In that study, her happy and sad facial (static) expressions were rated as one of the three most recognizable for the emotions (greatest distance between the ratings for her neutral vs. positive/negative expressions). We recruited the same woman for recording the videos of the present study.

##### 2.1.2.2. Experimental sentences

We constructed 16 unambiguous non-canonical object-verb-subject sentences in German. The experimental sentences had the following structure: NP1_masculine accusative case, patient_–verb–adverb_positive emotional valence_–NP2_nominative case, agent_ For example, *Den Marienkäfer kitzelt vergnügt der Kater*, lit. transl.: “The ladybug _NP1:Patient−Object−Acc_ tickles _verb_ happily _adverb_ the cat _NP2:Agent−Subject−Nom_.” The second author recorded all of the experimental sentences with neutral intonation at normal speed but with slight pauses to facilitate cue-pointing the on- and offsets of sentence regions for later analyses. The same speaker recorded comprehension questions in the active or passive voice (e.g., “Who is doing [previously named action] to [previously named patient]”; “Who is being [previously named action]?”). Only passive questions were used for the critical items (*N* = 16 questions). The passive-voice questions were motivated by initial findings (from another set of studies in the first author's Ph.D. thesis) that young adults were at ceiling for active-voice questions. To avoid ceiling-effects in the young adult group, we used passive-voice questions in the present experiments.

##### 2.1.2.3. Experimental scenes

For each sentence, we created a scene using Adobe Illustrator and commercially available clipart characters (animals and humans) and objects, yielding 16 (scene-sentence) item pairs. For the critical items, the target scenes (*N* = 16) consisted of 3 clipart characters and varied as to whether action were depicted or not (see Figure 2 for an example of the scene depicting action events). The middle character was always the patient of the action (the referent of NP1, henceforth: patient) and the outer two were the competitor and target agents. When no actions were depicted, only the three characters were visible. For half of the scenes, however, the competitor agent performed an action toward the patient but was not mentioned in the sentence. The target agent was the agent of the verb action mentioned in the sentence, i.e., the referent of NP2, henceforth: agent. The agent's facial expression was always happy in the experimental sentences. It hence matched in valence the speaker's positive but mismatched with her negative emotional facial expression. The patient had a neutral and the competitor had a negatively valenced facial expression. The competitor hence matched in valence with the speaker's negative but mismatched with her positive emotional facial expression. For a list of experimental sentences and scenes, see Appendices A.1 and C.2 in Münster (2016), see https://pub.uni-bielefeld.de/record/2906648.

##### 2.1.2.4. Pretest of the experimental items

We pre-tested the 16 experimental items with a sample of 4- to 5-year-old children (*N* = 20, *M*_age_ = 4.8) who did not take part in the eye-tracking study. Ten children were asked to point to the characters and the actions (e.g., “Who is the cat?” “Who is tickling the ladybug here?”). Character naming (scenes showed only the three characters) and action naming (scenes showed the three characters and their actions) were blocked (character naming before action identification). The children correctly identified the characters (96.9%) and actions (88.5%). Moreover, we tested the valence of the adverb in the sentences as cue to the agent. Ten additional children were asked to answer questions such as “Who tickles happily the girl?” (literal translation); “Who happily tickles the girl”? (idiomatic translation). Unlike in the experimental scenes both agent and competitor were depicted as performing the same action; only the agent smiled, permitting in principle agent identification. Critical item scenes were mixed with filler pictures and sentences that conveyed a negative or neutral valence. In 89.4% of the cases, children reliably identified the smiling agent when prompted with the adverb in the question, suggesting they can link the positive adverbs to the agent's smile.

##### 2.1.2.5. Fillers

In addition to the experimental items, we constructed filler items (*N* = 28, see Appendix A, recorded by PK). We wanted to avoid introducing clear biases regarding the manipulated variables via the fillers and for that reason balanced the assignment of valence and action depiction. In half of the fillers, one of the characters was depicted as performing the mentioned action (*N* = 14); the other half of the fillers had no actions (*N* = 14). For a given list, we presented a negative speaker prime face for one half of the fillers and a positive speaker prime face for the other half.

We further approximately balanced sentence valence. Twelve filler sentences had neutral verbs and adverbs (of which 4 were OVS) and 16 were positively valenced (SVO). We refrained from creating negatively valenced filler sentences due to ethical concerns regarding the children. We wanted a few more trials with SVO than OVS structure since SVO is more common in German. As a result, we had 24 fillers in SVO and 4 in OVS order, as well as 16 critical items in OVS order.

The 28 filler scenes consisted of clipart animals and humans. The characters either faced each other, faced the observer, or were depicted as looking in opposite directions to prevent participants from guessing who would be interacting with whom. For the fillers with two characters, both had a neutral facial expression (*N* = 20); for the fillers with three characters each had a different (negative, positive, and neutral) facial expression (*N* = 8). At least one of the filler characters in the scene also had a positive facial expression when the filler sentence was positive (*N* = 16). When the filler sentence was neutral (*N* = 12), at least one character's facial expressions was neutral or even negative, thus matching the negative emotional facial expression of the speaker but mismatching her positive expression. Since we refrained from creating negatively valenced filler sentences (ethical concerns regarding the children), we did not fully match negative prime faces with negative filler character faces and negative sentence valence.

#### 2.1.3. Design

Materials and design were identical for all three experiments. A 2 prime face (sentence-matching positive prime face vs. sentence-mismatching negative prime face) x 2 action (depicted action vs. no action) design yielded 4 conditions that combine with a constant target sentence (see Table 1). The prime face consisted of a short video of a woman's facial expression changing naturally from neutral into a broad and open smile or into a sad expression. Only the smile matched the sentence valence and the facial expression of the agent (see Table 1).

**Table 1:** Experimental conditions.

**Condition**	**Prime face**	**Action**
a	Positive (sentence-match)	Action
b	Positive (sentence-match)	No action
c	Negative (sentence-mismatch)	Action
d	Negative (sentence-mismatch)	No action

*The emotional valence of the speaker's prime face was either positive (happy), matching sentence valence, or negative (sad), mismatching sentence valence. Scenes either depicted actions on the patient or not*.

We created 4 base lists using a Latin Square, so that each participant encountered all conditions but each sentence in only one of the 4 conditions (Table 1). To counterbalance the position of the agent and competitor, we mirrored each critical scene. Adding the counterbalancing to the base lists, we obtained 8 lists. Agents and competitors were equally often on the right and on the left side of a scene for the experimental items. Moreover, in each list, 16 of the comprehension questions were in the active, 16 in the passive voice, and 12 were about facial emotions (“How are they feeling” when inspecting the faces); but critical items were all paired with a passive question. For the filler sentences, 16 questions were in the active voice, and 12 were about the facial emotion of the speaker and characters. We added all of the filler trials to each list, and pseudorandomized each lists such that two critical items never followed another. To counteract strategy building and order effects, trial presentation order was pseudo-randomized for each participant.

#### 2.1.4. Procedure

Participants first read an information sheet (or instructions were given orally) and gave written informed consent. For children, we obtained written consent from the parents beforehand and asked the children at the time of testing if they would like to take part. Participants were seated in front of the eye tracker (Eye-Link 1000 Eye tracker, SR Research, Ontario, Canada, remote setup).

In the instructions, we informed participants that they would see a speaker describe a scene with clipart characters. They were asked to concentrate on everything they see and to listen closely to the sentence because they would have to answer a question after each trial. The experiment started with a manual 5-point calibration and validation, repeated as necessary during the experiment. After successful calibration and validation, participants completed four practice trials. Each trial started with a fixation dot followed by a prime face video, in which the speaker's expression either changed from a neutral to a happy or sad looking face, see Figure 1, presented for 5,500 ms. The prime face was accompanied by the spoken phrase “Look!” to attract participants' (children's) attention. Note that 2,000 ms after the onset of the scene the sentence was played. In the scene, the characters were either shown as performing an action (depicted action condition) or as not performing an action (no action condition, see Figure 2). The scene in the no action condition was identical to the scene that appeared 500 ms after sentence end, i.e., the question screen. In this question screen, all depicted actions (if present) were removed and participants orally answered a question about who is doing what to whom (e.g., “Who is being tickled?”).

After 12 of the 28 filler trials participants were asked for the feelings portrayed by the speaker's face and one of the characters (i.e., “How are they feeling?”): Participants inspected a photo of the facial expression of the woman (the same valence as the prime face) next to the face of one of the characters from the previous scene. Additionally, after four of the critical trials (one per condition), the experimenter asked the participant to recall the facial expression of the speaker's face. We included these questions to also highlight the importance of the emotional faces in the study without providing explicit emotion labels, and to counteract a possible task bias of the prominent “who-does-what-to-whom” comprehension questions. These additional questions were counterbalanced such that across lists, participants were asked after each item in each experimental condition. Participants had no time limit, responded orally, and the experimenter wrote down the answer. An experiment session for one participant took (depending on the age group) approximately 30–60 min.

#### 2.1.5. Analysis

##### 2.1.5.1. Eye movements

The eye-movement analyses included the data from all critical trials (correct and incorrect responses). Analyzing only the eye movements from the correctly answered trials, especially for the children's data, would have led to loss of power due to an expected high error rate for the children and hence fewer data points, increasing the risks of Type II errors. For the analysis, scene areas of interest were the agent (the cat) and the competitor (the rat, Figure 2). Sentences were divided into NP1, verb, adverb, combined verb-adverb, and a prolonged NP2 (plus 500 ms to capture end of sentence effects) word region, as well as a region spanning from NP1 onset until 500 ms after the end of NP2 (“long region”), see Table 2 for mean duration. The word regions were determined from word onset of one word region until word onset of the next word region. The critical sentence regions were the verb and adverb: The verb is the first point in time when participants can discriminate the agent of the sentence if actions are depicted. The adverb region explicitly gives away the valence of the sentence and is thus the first point in time when the prime face could influence participants' attention. To capture spillover effects from the verb to the adverb, we also included a verb+adverb region in the analysis. We included a long region spanning the sentence to capture potentially pervasive effects. The NP1 region was analyzed to ensure that there is no fixation bias toward the target agent (vs. the competitor) before the verb.

**Table 2:** Mean duration of analyzed word regions in milliseconds.

	**NP1**	**Verb**	**Adverb**	**Verb–adverb**	**NP2+500 ms**	**Long region**
Mean dur.	1,017	770	894	2,039	1,342	5,045

For the analyses, we used the log-gaze probability ratio aggregated over participants and items. This is the log of the ratio of the probability of looking at the agent over the probability of looking at the competitor (ln(p(agent)/p(competitor))) (Arai et al., 2007; Carminati and Knoeferle, 2013). Since the log of 0 is undefined, a constant of 0.1 was added (to counteract missing data points regarding fixations to the agent or the competitor). The log probability ratio expresses the strength of the visual attention bias toward the agent relative to the competitor, and is symmetrical around zero. Zero indicates an equal amount of looks toward agent and competitor. A positive log ratio indicates a preference to inspect the agent over the competitor. A negative log ratio indicates a preference to inspect the competitor over the agent.

**Figure 3:**
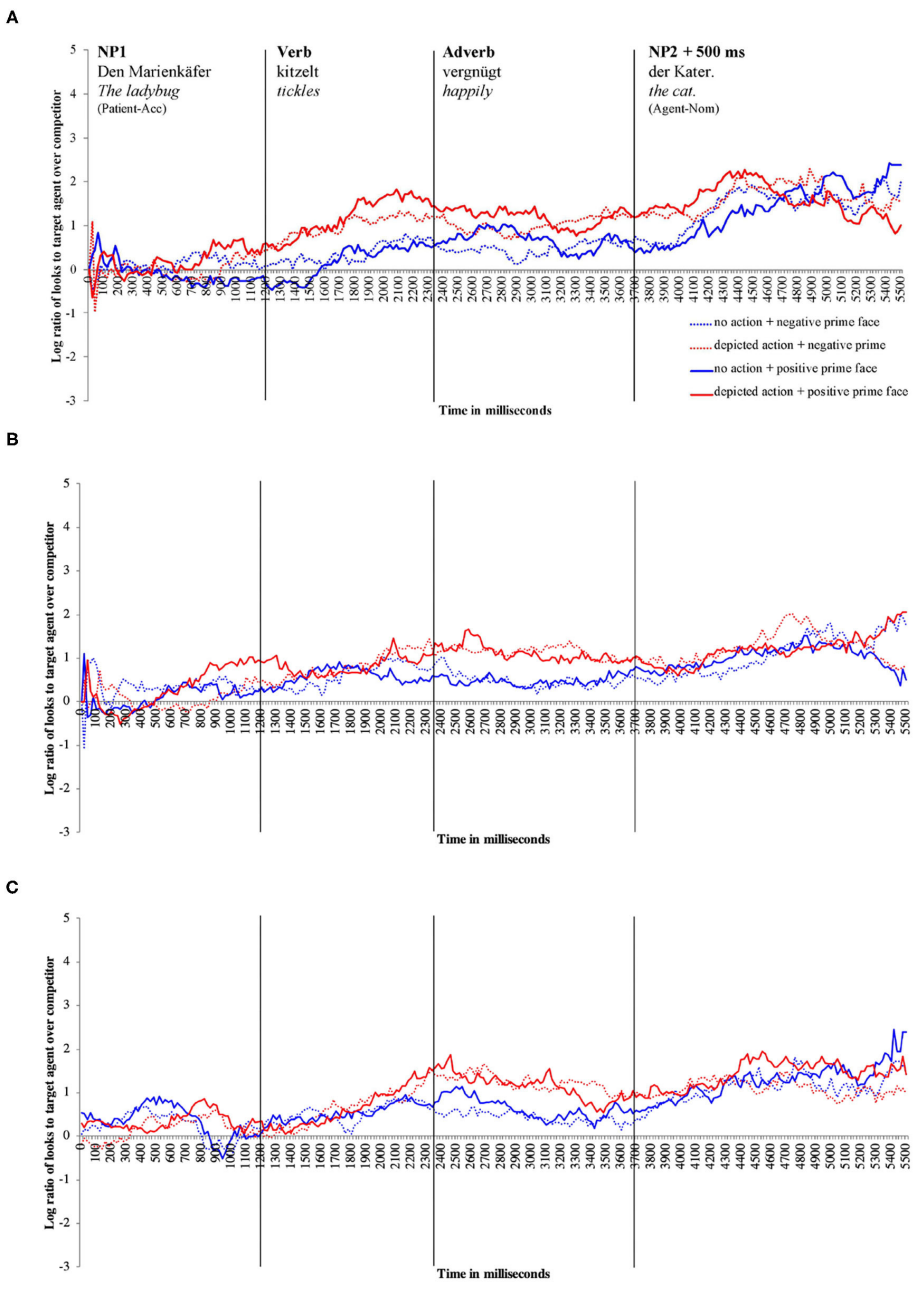
Time course graphs for **(A)** younger adults, **(B)** children, **(C)** older adults in milliseconds across the sentence. The solid lines indicate fixations in the positive prime face conditions, and the dotted lines indicate fixations in the negative prime face conditions. The red lines indicate fixations in the depicted action conditions, and the blue lines indicate fixations in the no-action conditions. Values above 0 indicate a preference to fixate the agent, and values below 0 indicate a preference to fixate the competitor.

For descriptive presentation, time course graphs plot the mean log gaze probability ratios (henceforth “log ratios”) computed on successive 20 ms time slots. The log ratios are plotted as a function of prime face and action depiction (see Figures 3A–C).

For the inferential analyses, log ratios were subjected to linear mixed effects models (using lmer of the lme4 package of R, Bates et al., 2015b) with action depiction (no action vs. depicted action), prime face (sentence-matching positive vs. sentence-mismatching negative) and age (children vs. younger adults vs. older adults) as fixed factors and participants and items as random intercepts. Note that age was only used as a factor in the analysis including all three age groups. The independent factors were centered (to avoid collinearity) and sum-coded. Random slopes for action and prime face were included in the subject random effect structure. Random slopes for action, prime face, and age were included in the item random effect structure. We are reporting the model results for the most parsimonious model obtained by reducing the random effect structure following Bates et al. (2015a). The *p*-values were obtained using Satterthwaite approximation (cf., Luke, 2017).

We first analyzed the data for each age group separately, using action and prime face as fixed factors. To further investigate how age modulates the action and prime face effects, we additionally conducted analyses for all three studies together, using age as a fixed factor in addition to action and prime face. Contrasts were set to compare children with younger adults and older with younger adults. The results for the verb, adverb, and verb-adverb regions were not corrected for multiple comparisons, since we had a priory hypotheses. By contrast, for the NP1, NP2, and long region, for which our hypotheses were less specific, results that would no longer be reliable after correcting for multiple comparisons will be indicated in a footnote. Note also that the studies were conceptualized as separate studies and analyzed separately using traditional F1 and F2 ANOVAs (PhD dissertation, Münster, 2016). We reference results that differ between these analyses in footnotes. The main findings, result patterns, and conclusions hold up across these analyses and when corrected for multiple comparisons.

##### 2.1.5.2. Accuracy data

Accuracy scores for the comprehension question (*N*= 640 per experiment) were subjected to generalized linear mixed models (using glmer of the lme4 package of R, Bates et al., 2015b). In all models, “Family” was set to “binomial” due to the categorical nature of the data. Note that the face-recall questions were not analyzed inferentially, since they yielded only 4 data points per participant, i.e., one for each condition. Children answered 82.5% of all face-recall questions correctly. Older adults recalled the speaker's facial expression correctly in 91% of cases and younger adults in 99% of cases. The filler questions asking about the feeling of the speaker's prime face and one of the scene characters were not analyzed, since their main function was to highlight the importance to attend to the emotional expressions relative to the post-trial who-does-what-to-whom questions in the study.

#### 2.1.6. Eye-Tracking Hypotheses

##### 2.1.6.1. Action effects and prime face effects

We predicted facilitation in processing the OVS sentence from the verb onwards when an action was (vs. was not) depicted (see Table 1 for conditions, Knoeferle et al., 2005; Zhang and Knoeferle, 2012). Participants should fixate the agent (cat) more than the competitor (the rat in Figure 2) in the depicted-action than the no-action conditions. These fixations should be anticipatory (i.e., emerge before the mention of the agent). If the effects of the action are pervasive, they should persist following the verb.

If the positive prime face facilitates processing of positive non-canonical (OVS) sentences, participants should launch more fixations (anticipatory or not) toward the agent (vs. competitor) in the positive (vs. negative) prime condition. Since the adverb gave away sentence valence, the earliest prime face effects should emerge in this region, and continue, if pervasive. Alternatively, effects of prime face may not generalize from priming an entire event to an agent within an event. They may also not generalize from subject-first sentences (Carminati and Knoeferle, 2013) to object-initial sentences (to the extent that the cognitive demands of processing object-initial sentences preclude the integration of speaker face with the adverb and the agent's facial expression).

Comparing effects of the two manipulated factors, we postulated a non-referential link between the speaker's smile, the adverb *happy* (pointing to the smiling agent), and the agent (smiling) in contrast to the referential link between a verb, its action, and the associated agent. Recall that referential relations have been shown to elicit more attention to objects than non-referential world language relations (Cooper, 1974). We accordingly predicted more pervasive and perhaps clearer action (depicted action vs. no action) than prime face effects (sentence-matching positive vs. sentence-mismatching negative).

##### 2.1.6.2. Lifespan effects

Delayed visual context effects (regardless of prime or action condition) might arise considering older adults' cognitive decline (e.g., Just and Carpenter, 1992) and children's processing difficulties with non-canonical sentences (e.g., Dittmar et al., 2008), reflecting perhaps resource limitations. However, Carminati and Knoeferle (2013) indicated that older and younger adults seem to inspect valenced events with comparable timing when primed by a speaker's happy or sad expression. They differed, however, by valence. Older (vs. younger) adults benefited more from a smiling prime face for anticipating a positive (vs. negative) event; younger (vs. older) adults benefited more from a negative face-sentence valence match. For the present experiment, this asymmetry predicts stronger face-priming effects in older than younger adults since prime face and sentence matched in positive valence only. For children, non-referential context effects are not expected under at least some accounts (see Knoeferle and Crocker, 2006, p. 524f for discussion of non-referential context effects in kindergarten children).

#### 2.1.7. Accuracy Hypotheses

If the depicted actions facilitate comprehension off-line, participants' response accuracy should be higher when an action was (vs. was not) depicted. If the same is true for the positive prime face, then participants' accuracy should be higher when the prime face matched (vs. mismatched) sentence valence. If the action cues the agent more effectively than the prime face, accuracy should be higher for action presence than speaker prime face match. Regarding lifetime effects: If the comprehension questions are easy for adults (younger and older), accuracy should be high and any differences between the conditions should be negligible (ceiling effects). For children, however, we expected a boost in correct responses when a depicted action (vs. no action) illustrated the thematic role relations (cf., Zhang and Knoeferle, 2012). Since the speaker's smile cues the agent only indirectly (and not via verb reference like the actions), 4- to 5-year-old children might not be able to use this cue, predicting a null effect of speaker prime.

### 2.2. Results

#### 2.2.1. Experiment 1: Younger Adults

##### 2.2.1.1. Eye movements

Figure 3A shows the time course graph for younger adults. Descriptively, younger adults fixate the agent more than the competitor when an action is (vs. isn't) depicted as soon as the verb information becomes available and continues until the end of the sentence. By comparison, the difference between the positive and negative prime face conditions appears less clear. However, during the verb–adverb region, the solid lines deviate from the dotted lines, indicating a higher probability of looks to the agent in the positive vs. the negative prime face conditions.

**Figure 4:**
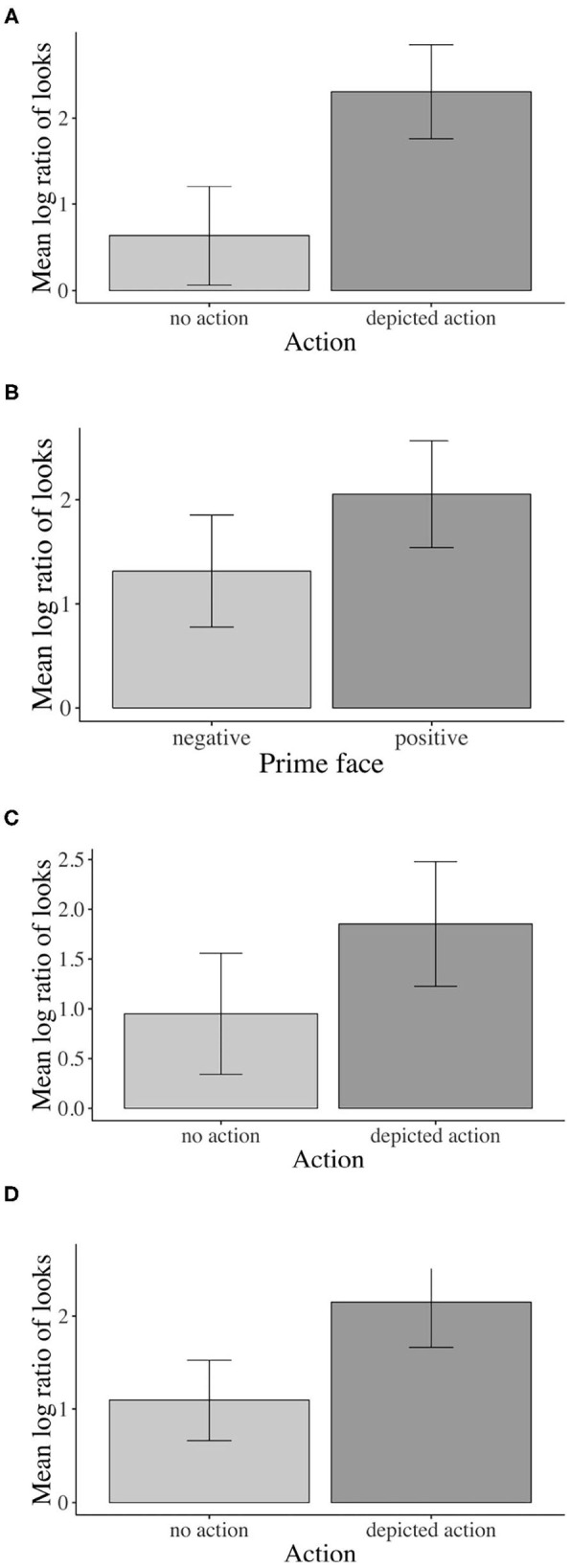
Eye-tracking results for main effects of action and prime face. Younger adults: **(A)** Sign. main effect of action in the verb region, **(B)** Sign. main effect of prime face in verb–adverb region. Children: **(C)** Sign. main effect of action in the verb region. Older adults: **(D)** Sign. main effect of action in verb–adverb region. Error bars show 95% confidence intervals.

Inferential statistics for younger adults indicated a main effect of action: Younger adults fixated the agent significantly more than the competitor during all analyzed word regions when an action was (vs. was not) depicted, except for the NP1 region (i.e., verb region^1^: β = –0.826, SE = 0.1409, df = 577.3, *t* = –5.867, *p* < 0.01, see Figure 4A).

Moreover, a main effect of prime face emerged in the verb–adverb (β = –0.364, SE = 0.129, df = 522.3, *t* = –2.810, *p* < 0.01, see Figure 4B) and in the long region (β = –0.224, SE = 0.106, df = 572.7, *t* = –2.100, *p* < 0.05)^2^. Younger adults fixated the agent significantly more than the competitor when the speaker's prime face was positive compared to when it was negative. The interaction between action and prime face was not significant (e.g., long region: β = 0.08385, SE = 0.106, df = 572.8, *t* = 0.785, *p* = 0.432).

##### 2.2.1.2. Accuracy

Participants answered 96% of all questions correctly across conditions (range: 92–98%). The analysis yielded no significant effects of prime face or action for younger adults^3^.

#### 2.2.2. Experiment 2: Children

##### 2.2.2.1. Eye movements

Children fixated the agent more than the competitor when an action was (vs. was not) depicted; this relative increase in attention to the agent when actions were depicted started late during the verb and was strongest during the adverb region (see Figure 3B). Gaze in the positive and negative prime face conditions did not differ much, indicating no modulation of agent vs. competitor fixations by prime face.

For children, like for younger adults, the inferential analysis indicated a main effect of action in all word regions except for the NP1 region (verb–adverb: β = –0.547, SE = 0.166, df = 38.3, *t* = –3.294, *p* < 0.01): Children fixated the agent more than the competitor when an action was (vs. was not) depicted. However, in the verb region, the action effect just reached the conventional significance threshold of α = 0.05, i.e., β = –0.453, SE = 0.212, df = 17.255, *t* = –2.133, *p* = 0.047, see Figure 4C)^4^^,^ ^5^. Neither the effect of the emotional prime face nor the interaction between prime face and action reached significance in any of the word regions.

##### 2.2.2.2. Accuracy

**Figure 5:**
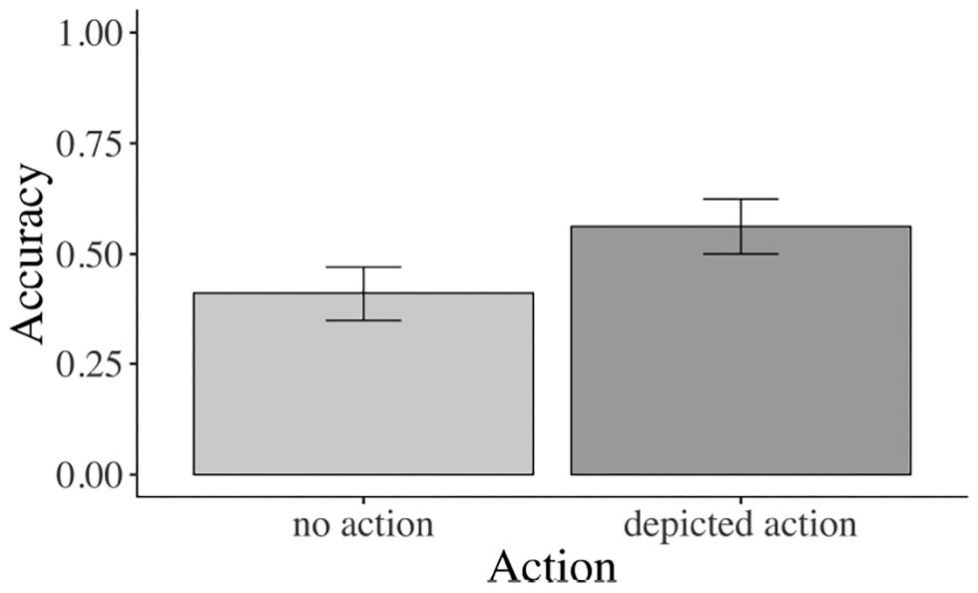
Accuracy results for main effect of action. Children: Sign. main effect of action for accuracy. Error bars show 95% confidence intervals.

Children answered significantly more comprehension questions correctly when an action was (56 vs. 41% was not) depicted (β = –0.477, SE = 0.149, z = –3.195, *p* < 0.01, see Figure 5). Neither the effect of prime face nor the prime face by action interaction was significant.

#### 2.2.3. Experiment 3: Older Adults

##### 2.2.3.1. Eye movements

Descriptively, older adults fixated the agent (vs. competitor) more when actions were (vs. were not) depicted (see Figure 3C). As the time course graph for older adults shows, this pattern started late during the verb, lasting through the adverb region. In the NP2 region and until sentence end, action presence no longer modulated fixation behavior. The prime face did not seem to affect older adults' fixations (the solid and dotted lines do not deviate).

Corroborating the result pattern that we see descriptively in the time course graph, when actions were (vs. were not) depicted, older adults fixated the agent significantly more than the competitor in the combined verb–adverb (β = –0.523, SE = 0.200, df = 15.0, *t* = –2.605, *p* = 0.019, see Figure 4D) but also in the adverb (β = –0.688, SE = 0.216, df = 15.0, *t* = –3.174, *p* < 0.01) region. The main effect of action was not significant in the verb region alone and in any other word region. No other significant effects involving the independent factors emerged.

##### 2.2.3.2. Accuracy

Older adults answered 89% of all questions correctly across all conditions (range: 84–93%), with no significant effects of the manipulated factors^6^.

#### 2.2.4. Across Age Groups

##### 2.2.4.1. Eye movements

The main effect of action was significant in all analyzed word regions (e.g., adverb: β = –0.662, SE = 0.116, df = 16.7, *t* = –5.700, *p* < 0.01), except for NP1. Participants in all age groups fixated the agent significantly more than the competitor when actions were (vs. were not) depicted.

In the verb region, a significant action by age interaction^7^ (β = 0.291, SE = 0.134, df = 156.2, *t* = 2.172, *p* < 0.05) confirmed that the inspection of the agent (vs. competitor) varied with comprehender age group.

**Figure 6:**
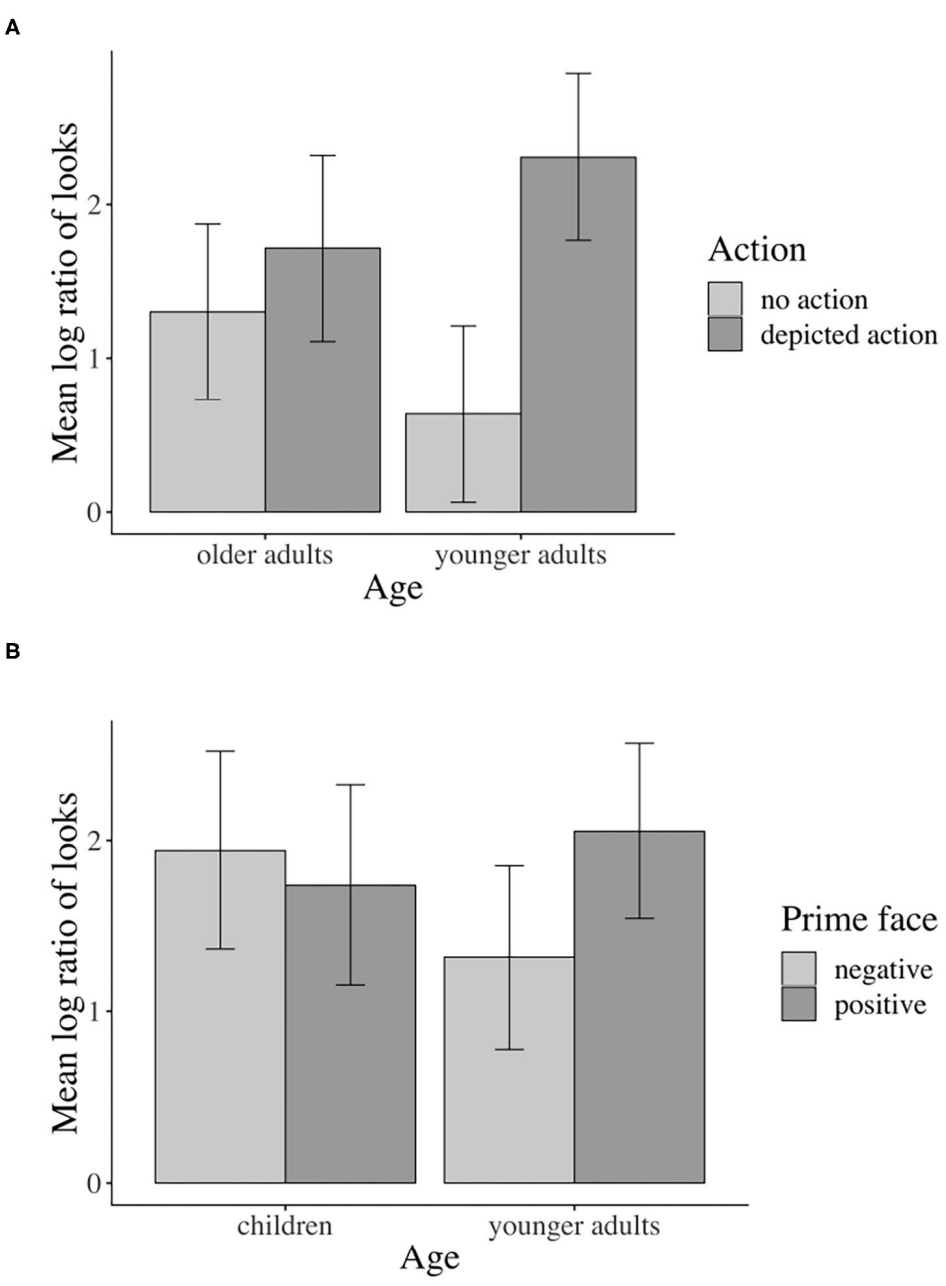
Eye-tracking interactions by age: **(A)** Sign. action x age interaction for younger vs. older adults in the verb region. **(B)** Sign. prime face x age interaction for younger adults vs. children in the verb–adverb region. Error bars show 95% confidence intervals.

Following the significant interaction of action with age (all three groups), we conducted subset interactions (children vs. younger adults; older vs. younger adults) of which only the interaction including older and younger adults was reliable. For younger adults only, the contrast of depicted action vs. no action was significant (β = –1.653, SE = 0.386, df = 29.1, *t* = –4.278, *p* < 0.01, Bonferroni-adjusted; older adults: n.s.). No other comparisons were significant (see Figure 6A).

In the verb–adverb region, the prime face by age interaction was only just significant (β = 0.217, SE = 0.108, df = 1739.9, *t* = 2.000, *p* < 0.05)^8^. As Figure 6B indicates, in the verb–adverb region, younger adults but not children fixated the agent more than the competitor when the prime face was positive (vs. negative). No other significant effects involving the independent factors emerged.

##### 2.2.4.2. Accuracy

**Figure 7:**
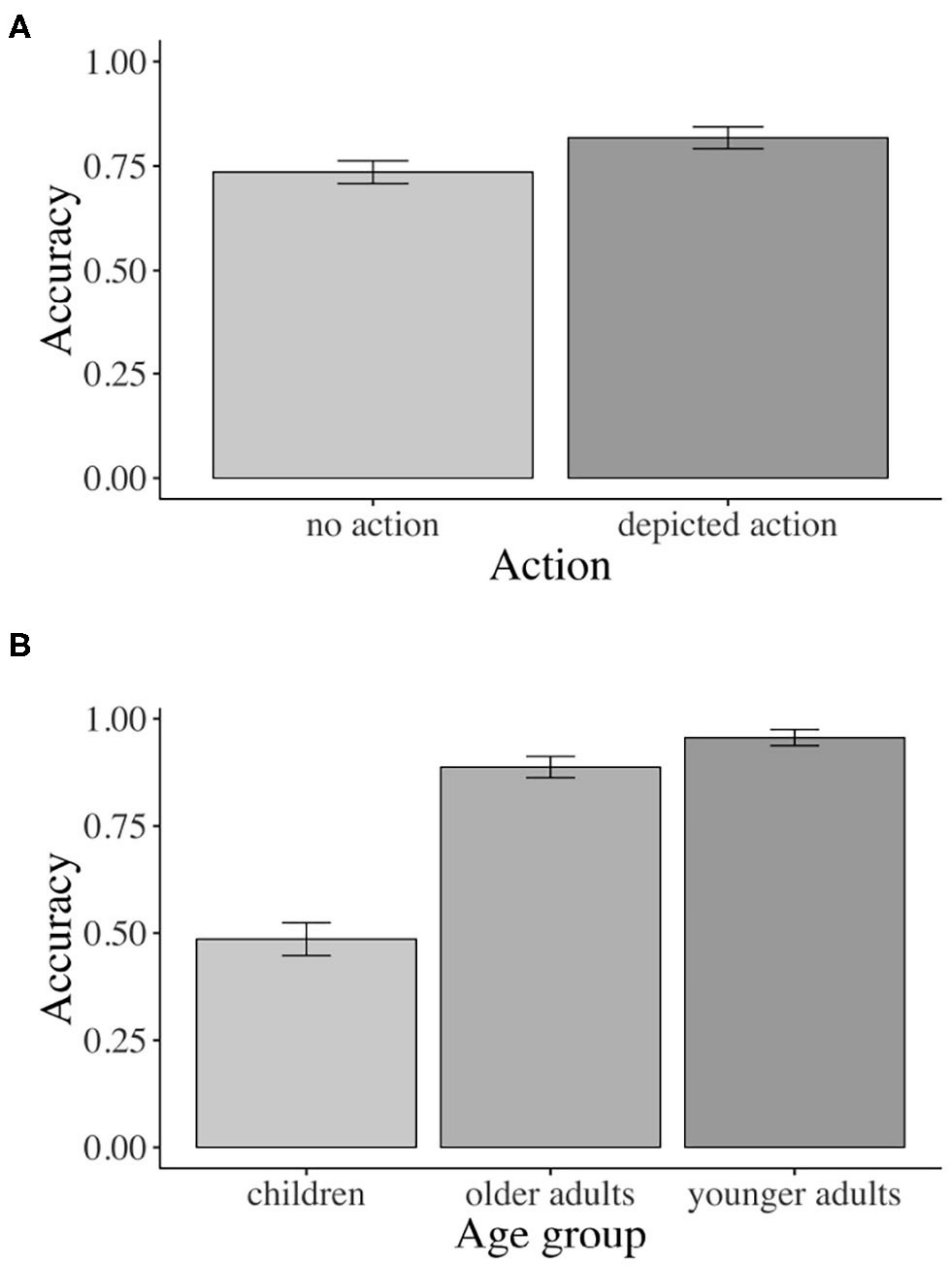
Accuracy: **(A)** Sign. main effect of action across age groups. **(B)** Sign. main effect of age. Error bars show 95% confidence intervals.

The accuracy analysis yielded a significant main effect of action (β = –0.358, SE = 0.089, z = –4.007, *p* < 0.01). Across all three age groups, participants' comprehension question accuracy was significantly higher when an action was (82 vs. 74% was not) depicted (see Figure 7A).

Additionally, the results yielded a significant main effect of age for both age contrasts (children vs. younger adults: β = –1.827, SE = 0.101, *t* = –18.024 *p* < 0.01; older vs. younger adults: β = 0.354, SE = 0.117, *t* = 3.024, *p* < 0.01). As Figure 7B shows, children (49%) and older adults (89%) answered significantly fewer comprehension questions correctly than younger adults (96%) across conditions.

## 3. General Discussion

In three visual-world eye-tracking experiments, we compared the effects of referential and non-referential world–language relations on children's, younger, and older adults' thematic role assignment. Participants in each age group first saw a speaker smiling or looking sad. Next, in the depicted action condition, they inspected a clipart scene depicting a ladybug being tickled by a happy-looking cat and arrested by a grumpy-looking rat (Note that this is one example out of 16 critical trials). In the no-action condition, the scene showed the characters side by side but they did not perform any actions. While participants inspected the scene, they listened to a German sentence. That sentence had an object-first word order (which is non-canonical but grammatical in German) and mentioned the happy tickling event (e.g., “The ladybug _NP1:Patient−Object−Acc_ tickles _verb_ happily _adverb_ the cat _NP2:Agent−Subject−Nom_”). We assessed—by measuring looks to the happy-looking cat vs. the grumpy-looking rat—to what extent participants would anticipate the cat (filling the subject and agent role) before its mention. Such anticipation could be informed by the verb, referring to the cat's tickling action, and/or by the speaker's smile matching the adverb “happy” and the smile of the cat.

Analyses of the looks corroborated that the referenced action lead to anticipation of its agent (cat) in all age groups. When an action was (vs. was not) depicted, children, young, and older adults looked more at the happy-looking cat, engaged in tickling (than the grumpy-looking rat, engaged in arresting). For young adults, this pattern emerged robustly at “tickles,” replicating previous findings (cf., Knoeferle, 2007; Zhang and Knoeferle, 2012). For older adults and children, it emerged later, during “tickles happily” and “happily” (see Wassenaar and Hagoort, 2007; Zhang and Knoeferle, 2012 on age delays in situated language comprehension, and DeLong et al., 2012 on comprehension in strictly linguistic contexts). Processing non-canonical (object-first) sentences imposes a high cognitive load even in young adults (Matzke et al., 2002). For children compared to younger adults, the associated high load may have delayed agent anticipation.

These real-time results were corroborated by the accuracy data: children answered more comprehension questions correctly when an action was vs. was not depicted i.e., no actions: 41% vs. depicted actions: 56% correct comprehension-question responses). However, even in the depicted action condition, children's correct answers were not significantly different from chance. This is not surprising given the age of the children and what we know about their comprehension of case marking and difficulty with non-canonical object–verb–subject sentences (Dittmar et al., 2008) and may point to a limited effect of the action depiction. Nonetheless, compared with the no-action conditions, the depicted action conditions elicited a boost of 15% in accuracy, corroborating effects of the visual context on comprehension. The younger adults (vs. older adults and children) gave more correct answers across conditions, in line with the view that this age group was at the height of linguistic and cognitive abilities (see Figure 7B).

The speaker's positive (vs. negative) face affected younger but not children's and older adults' on-the-fly processing. The younger adults had a higher probability of looking at the smiling cat (vs. the grumpy rat) when the speaker's facial expression was happy (vs. sad). This gaze pattern emerged during “tickles happily” and was hence anticipatory. Emotional facial expressions can thus rapidly facilitate young adults' sentence processing (Carminati and Knoeferle, 2013) even for non-canonical sentences and the anticipation of an agent within an event. Younger adults must at least have recognized and interpreted the speaker's emotional facial expression; they kept it in memory while interpreting the utterance, and linked it to the emotionally matching adverb, eliciting in turn anticipation of the smiling cat. The high accuracy of 96% for comprehension questions rules out that the anticipation occurred without comprehension of the object-first sentences. The absence of agent anticipation before the verb is compatible with the view that “happily” prompted participants' to link the speaker's smile with the agent's smile.

Children and older adults may have managed to keep the speaker's smile in working memory (as suggested by high accuracy in the face-recall task), but perhaps that representation was not sufficiently active to match it with “happily” and the agent's smile. Alternatively, or in addition, linking a speaker's emotional facial expression, with the valence-matching adverb and the agent's smile may have been cognitively demanding for older adults and 4- to 5-year-olds, precluding on-the-fly effects on agent anticipation.

An alternative interpretation of the prime face effect is that language did not enable agent anticipation. Instead, anticipation was elicited by visual similarity between the speaker and agent faces (both smiling). However, we think this is unlikely for the following reasons: Participants previewed the target scene (2,000 ms) before the sentence started (see Figure 2). If the prime face effects were based on visual similarity alone, we should see priming effects from the happy-looking prime face to the happy-looking target agent during preview. Additional analyses revealed no significantly higher probability of looks to the happy-looking agent (vs. grumpy-looking patient) after a happy-looking (vs. sad-looking) speaker face. Moreover, no prime face effects emerged before the verb. Thus, the prime face effect that emerged during the verb–adverb region for younger adults depends also on verb and adverb meaning and not just on visual similarity. Furthermore, our pretest (see section 2.1.2.4) confirmed that, offline, even 4- to 5-year-old children linked the positive adverb to the target agent's happy face. The face recall questions during the eye-tracking experiment revealed that all age groups kept the prime face valence in memory for the duration of the trial and could label the speakers facial expressions correctly. The latter points suggest that (a) participants interpreted the smiling speaker face and the target agent as similar and (b) keeping the speaker's smile in memory was task relevant.

Our findings show that verb–action relations and non-referential emotional world–language relations seem to inform real-time language processing to different degrees (see Knoeferle, 2019 for a comprehensive review). Action effects were pervasive in all age groups; the speaker's emotional prime face only facilitated younger adults' sentence processing. Further, younger adults showed an action effect in all analyzed word regions except for the NP1 region, while the speaker's prime face effect was only significant in the verb–adverb region and across the sentence. Across age groups, participants answered significantly more off-line comprehension questions correctly when an action was (vs. was not) depicted; for the speaker's emotional face, no off-line effects emerged. These differences underscore that the examined referential and non-referential world-language relations influence comprehension in distinct ways.

Perhaps the differences in action compared with face prime effects are due to the serial presentation of the prime face (vs. concurrent presentation of the actions). Unlike the speaker's face, the action depictions need not be kept in working memory but can be inspected during comprehension. However, similar depicted actions have had clear effects on comprehension even when presented prior to the sentence (Knoeferle and Crocker, 2006; Knoeferle et al., 2011, 2014).

The context effects reported in the present experiments highlight that we must better specify how distinct world–language relations are understood (Smaldino, 2019). Below we illustrate one such specification of the representations and mechanism that underpin distinct context effects in real-time language comprehension.

### 3.1. Accommodating the Results in the sCIA

The sCIA (Münster and Knoeferle, 2018) is a (not computationally- implemented) processing account that models the incremental interplay of sentence processing with the visual context via three steps: (i) incremental interpretation of language and associated expectations, (ii) language-mediated attention, and (iii) the integration of interpretation and visual context representations (for a computationally implemented version see Crocker et al., 2010). As an interpretation is built, it guides attention in context and to context representations (captured by “scene”: the speaker's face or voice, other characters, objects, and action events among others). Representations of language, and the scene are passed on via a working memory buffer. Variability of comprehension and attention (due to comprehender age, level of education and/or cognitive abilities) is accommodated via the properties of the comprehender, “ProCom,” and that variable can modulate (the activation of) mental representations and processes. Both comprehender properties and (non-linguistic, social) context can elicit expectations captured probabilistically in ants.

We exemplify how the sCIA accommodates variability in the main effects of depicted action and prime face during the verb–adverb region^9^. For that region, the main effect of action was significant for all age groups, whereas the main effect of prime face was significant for younger adults only^10^. Participants have already processed the positive prime face of the speaker and the account captures that representation via working memory. They have also interpreted *The ladybug*_Patient−Acc_ as the patient of the sentence *The ladybug*_NP1:Patient−Acc_ *tickles*_Verb_ *happily*_Adverb_ … and may have noticed a cat that is smiling and holding a feather in the direction of the ladybug, depicting tickling. The interpretation and happy event are represented in working memory, as is a rat, as looking grumpy and as performing an arresting action toward the ladybug.

Against this processing history, *tickles happily* is integrated into the existing interpretation. Following the verb, the account assumes a referential search for a tickling action, eliciting attention to its referent, with some attention also going to the nearby agent, the cat. Inspection of an agent receives further support from ant_s_^p^. This parameter captures in a probabilistic manner the expectations during comprehension, informed by linguistic and world knowledge. Following the patient-initial sentence beginning and transitive verb, ant_s_^p^ would elicit some anticipation of an agent. The identity of the agent remains unclear but can be narrowed down via the verb in relation to the action. The expectations and the interpretation are reconciled with the scene representations, eliciting more anticipation of the tickling cat than the rat when actions are depicted (main effect of action). The sCIA has an updated interpretation of a ladybug undergoing a tickling action. Age and associated differences in cognitive capacities can modulate these processes and representations (via ^p^ in ant_s_^p^). A lower ^p^-value is set by ProCom for children and older adults, yielding reduced attention to the cat (vs. rat) for children and older (compared with younger) adults (main effect of age).

The actions are co-present during comprehension but the speaker's positive facial expression is assumed to decay in the sCIA, differentially by age and associated cognitive resources (main effect of prime face in young adults and reliable age x prime face interaction across experiments). Decay of the speaker's smile might happen faster for children and older than for the young adults (activation of the face representations could be down-graded by ProCom for children and older adults). Partially or fully decayed face representations in these participant groups would lead to an absence of agent anticipation based on the face prime. Alternatively, children's and older adults' representations (stored in ant_s_^p^) are not sufficiently detailed or active. The non-referential link between the prime face, the positive adverb, and the target agent could create a cognitively demanding processing situation. In that situation, children and older adults—likely characterized by lower processing speed than younger adults—might fail to link the speaker's prime face to the verb–adverb in time, precluding agent anticipation^11^. Based on the current results, we cannot disentangle where the age differences in the face prime effect arise (as part of utterance-mediated attention, or when scene and sentence representations are integrated in the sCIA).

## 4. Conclusion

In three visual-world eye-tracking studies, we investigated the effects of a speaker's emotional facial expression and depicted action events on real-time language processing of 4- to 5-year-old children, 18- to 30-year-old, and 60- to 90-year-old adults. A speaker's emotional facial expression and depicted action events differed in the way and extent in which they modulated children's, younger, and older adults' visual attention and sentence processing. Acknowledging and investigating the impact of referential and non-referential world–language relations during comprehension across the lifespan is crucial given that psycholinguistic research aims to better understand the representations and mechanisms implicated in real-time language processing per empirical research and (computational/theoretical) modeling.

## Data Availability Statement

The datasets generated for this study are available on request to the corresponding author.

## Ethics Statement

The studies involving human participants were reviewed and approved by the Bielefeld University Ethics Committee (Vote 2013-007). Written informed consent to participate in this study was provided by the participants' legal guardian/next of kin. Written informed consent was obtained from the individual(s) for the publication of any potentially identifiable images or data included in this article.

## Author Contributions

PK and KM created the stimuli and designed the experiments, and wrote the manuscript. KM conducted the studies and analyzed the data. Analyses of these data have previously been published in Münster (2016) and in parts in Münster et al. (2015).

## Conflict of Interest

The authors declare that the research was conducted in the absence of any commercial or financial relationships that could be construed as a potential conflict of interest.

## Footnotes

^1^See Supplementary Material for model parameters of the other regions in which the main effect of action was significant.

^2^The alpha level after correction for multiple comparisons is 0.05/3 = 0.016 (two main effects and one interaction), the prime face effect in the long region does not hold up if the corrected alpha level is applied. The ANOVA analysis in Münster (2016) yielded marginal prime effects in these regions.

^3^The ANOVA analysis in Münster (2016) yielded a marginal main effect of action.

^4^The action effect in the NP2 region does not hold if a corrected alpha level of 0.016 is assumed.

^5^The ANOVA analysis in Münster (2016) yielded a significant main effect of action in the verb region but no significant action effect in the NP2 region.

^6^The ANOVA analysis in Münster (2016) yielded a marginal main effect of action.

^7^The ANOVA analysis in Münster (2016) yielded a marginal interaction, carried by differences for younger adults vs. children.

^8^The ANOVA analysis in Münster (2016) yielded a marginal interaction in the adverb region.

^9^Note that we are not disentangling the effects for the verb and adverb regions here, since the focus in this section is on the distinct world–language relations of the prime face and the depicted action and their distinct effects on language processing, not on their specific time course (for details, see section 2.2.4).

^10^Note that one of the current limitations of the sCIA is that ProCom still acts as a “black box,” that is, the account does not yet specify the interacting mechanisms between the experiential and biological properties of the comprehender: It makes no claims about whether one property might have a larger influence on processing than the other and how the different comprehender properties interact with each other during processing.

^11^Note, however, that we are not claiming that differences in cognitive abilities and/or reduced processing speed between younger adults and children/older adults are the (only) reasons for the age differences in our results. Differences in cognitive abilities and/or processing speed are only one possible interpretation and we are using this interpretation to exemplify the workflow of the sCIA.

## Appendix

### A Filler sentences

**German originals**

1. Den Flamingo besprüht soeben der Hai.
2. Den Eisbär befragt nachher der Frosch.
3. Den Stier malt lächelnd der Wurm.
4. Den Koala bekleckert kurzerhand die Gans.
5. Das Eichhörnchen erzählt einfühlsam der Maus eine Geschichte.
6. Der Mann streichelt sanft den Fuchs.
7. Das Schaf springt jubelnd zu dem Pferd.
8. Die Schildkröte verehrt fasziniert die Biene.
9. Die Tänzerin beklatscht glückselig den Clown.
10. Der Kapitän beguckt aufmerksam den Fisch.
11. Der Künstler bemalt demnächst den Hund.
12. Die Nonne segnet frohgemut den Butler.
13. Die Frau winkt höflich dem Mann zu.
14. Die Blondine spielt ein schönes Lied für die Eule.
15. Der Sohn bewundert seinen Vater und schreibt ihm ein Gedicht.
16. Das Känguru bewirtet rücksichtsvoll den Marder.
17. Die Hausfrau badet behutsam das Schwein.
18. Das Zebra pflegt sorgfältig den Engel.
19. Die Prinzessin herzt gefühlvoll den Zwerg.
20. Der Mönch ermuntert zaghaft das Dromedar.
21. Die Ente trinkt im Augenblick Wasser mit der Ameise.
22. Die Schlange beäugt mehrmals das Reh.
23. Die Fee liest morgens Zeitung mit dem Schneemann.
24. Der König begegnet tagsüber dem Weihnachtsmann.
25. Der Papagei beobachtet öfters den Bären.
26. Der Fuchs läuft nun auf den Sportler zu.
27. Der Wanderer verarztet gleich die Katze.
28. Der Käfer trocknet sogar den Hasen.

**English translation**

1. The shark is spraying the flamingo.
2. The frog later questions the polar bear.
3. The worm smilingly paints the bull.
4. The goose splotched the koala.
5. The squirrel sensitively tells the mouse a story.
6. The man gently strokes the fox.
7. The sheep jumps to the horse, cheering.
8. The turtle worships the bee, fascinated.
9. The dancer blissfully applauds the clown.
10. The captain looks attentively at the fish.
11. The artist soon paints the dog.
12. The nun happily blesses the butler.
13. The woman politely waves to the man.
14. The blonde plays a beautiful song for the owl.
15. The son admires his father and writes him a poem.
16. The kangaroo respectfully hosts the marten.
17. The housewife gently bathes the pig.
18. The zebra carefully nurses the angel.
19. The princess hugs the dwarf, with feeling.
20. The monk timidly encourages the dromedary.
21. The duck is drinking water with the ant.
22. The snake eyes the deer several times.
23. The fairy reads the newspaper with the snowman in the morning.
24. The king meets Santa Claus during the day.
25. The parrot watches the bear several times.
26. The fox is now running towards the sportsman.
27. The hiker immediately treats the cat.
28. The beetle even dries the hare.
