# Supplementary material for: Referential vs. Non-referential World-Language Relations: How Do They Modulate Language Comprehension in 4 to 5-Year-Olds, Younger, and Older Adults?
Source: Front Psychol. 2021 Jan 13;11:542091. doi: 10.3389/fpsyg.2020.542091 (PMC7838495; doi:10.3389/fpsyg.2020.542091)
Supplement: Supplementary file 1 [file Data_Sheet_1.PDF]

Supplementary Material (Maquate & Knoeferle, 2020)

Additional model parameters of significant main effects of action for the eye-tracking data by age group and word region.

Note: only additional model parameters that are not mentioned in the paper are reported here. Other effects and interactions that are not mentioned in the paper were not significant.

Younger adults

**Region**	**ß**	**SE**	**df**	**t**	***p***
Verb-adverb	-0.741	0.177	18.800	-4.186	<0.001
Adverb	-0.673	0.185	43.600	-3.636	<0.001
NP2	-0.502	0.129	574.600	-3.872	<0.001
Long region	-0.412	0.106	573.100	-3.858	<0.001

Children

**Region**	**ß**	**SE**	**df**	**t**	***p***
Adverb	-0.628	0.157	572.600	-4.000	<0.001
NP2	-0.330	0.150	573.000	-2.198	<0.05
Long region	-0.315	0.111	572.600	-2.826	<0.01

Across age groups

**Region**	**ß**	**SE**	**df**	**t**	***p***
Verb	-0.493	0.131	17.300	-3.743	<0.01
Verb-adverb	-0.603	0.112	14.900	-5.349	<0.001
NP2	-0.318	0.101	15.000	-3.140	<0.01
Long region	-0.317	0.088	14.700	-3.603	<0.01
